# STRYDE versus PRECICE magnetic internal lengthening nail for femur lengthening

**DOI:** 10.1007/s00402-021-03943-8

**Published:** 2021-05-13

**Authors:** Sherif Galal, Jonggu Shin, Peter Principe, Nathan Khabyeh-Hasbani, Rena Mehta, Amber Hamilton, S. Robert Rozbruch, Austin T. Fragomen

**Affiliations:** 1 Limb Lengthening and Complex Reconstruction Service, Department of Orthopaedic Surgery, Hospital for Special Surgery, Weill Medical College Cornell University, New York, NY USA; 2 Department of Orthopaedic Surgery, Kasr Al-Ainy School of Medicine, Cairo University, Cairo, Egypt

**Keywords:** Femur, Intramedullary, Lengthening, PRECICE^®^, STRYDE^®^

## Abstract

**Introduction:**

Magnetic internal lengthening nails (MILNs) have been used for femoral lengthening to avoid complications associated with external fixation. The titanium version of the MILN (PRECICE^®^) has been in use since 2011 but had limitations (50–75 lb) in post-operative weight bearing. A new stainless-steel version of the MILN (STRYDE^®^) allows 150–250 lb of post-operative weight bearing.

The aim is to compare the outcomes of using these two different MILNs for both unilateral and bilateral femoral lengthening.

**Methods:**

A single-center, retrospective cohort study was conducted in which patients’ records were reviewed from the period from January 2017 to March 2020. A total of 66 femoral lengthening procedures were included in the study and were divided into two groups: STRYDE^®^ group (30 femora) and PRECICE^®^ group (36 femora). Outcomes assessed were the 6-months post-operative Limb Deformity-Scoliosis Research Society (LD-SRS) Score, adjacent joint range of motion (ROM), average distraction rate, bone healing index (BHI), and complications.

**Results:**

No statistically significant difference was found between the two groups in regard to the (LD-SRS) score, hip ROM, or knee ROM. Statistically significant differences were found between the two groups in regard to BHI (average of 0.84 months/cm and 0.67 months/cm for STRYDE^®^ and PRECICE^®^, respectively) and distraction rate (average of 0.6 mm/day and 0.9 mm/day for STRYDE^®^ and PRECICE^®^, respectively).

No mechanical nail complications were reported in the STRYDE^®^ group compared to three events of nail failure in the PRECICE^®^ group. One femur in the PRECICE^®^ group needed BMAC injection for delayed healing compared to four femurs in the STRYDE^®^ group.

**Conclusion:**

The STRYDE^®^ MILN yields comparable functional results to those of PRECICE^®^ MILN and shows fewer mechanical nail complications. However, STRYDE^®^ MILN requires a slower distraction rate and yields slower healing (larger BHI).

**Level of evidence:**

Level III, Therapeutic study.

**Supplementary Information:**

The online version contains supplementary material available at 10.1007/s00402-021-03943-8.

## Introduction

Ilizarov laid the groundwork for successful limb lengthening using distraction osteogenesis with circular external fixation. Unfortunately, external fixation provokes an array of complications including pain, soft tissue tethering, pin site infections, neurovascular entrapment, limited joint motion, and prolonged times needed to wear the apparatus to achieve union [1–5]. Internal lengthening nails are being increasingly utilized to evade such complications and improve patients’ comfort level during the lengthening process [6–9].

The FITBONE^®^ nail [10] introduced the concept of motorized intramedullary lengthening nails, motor is powered by an electric current generated from a transducer implant under the skin.

The PRECICE^®^ magnetic internal lengthening nail (MILN), introduced in 2011, overcame key imperfections of the early intramedullary nails. This motorized implant employed a magnetic actuator drive mechanism with an internal rare earth magnet to distract the nail using an external electromagnetic activator [11]. This made it possible for surgeons to adhere to the Ilizarov method by maintaining control of the distraction rate and rhythm, a critical advantage of the PRECICE^®^ over its predecessors. The PRECICE^®^ MILN has revolutionized limb lengthening with an outstanding healing profile, high accuracy and precision, and low complication rates [12–16]. However, there remained a need for an improved implant that would allow greater weight bearing and less mechanical failure [17]. The STRYDE^®^ is the newest generation MILN that further improved intramedullary devices by increasing post-operative weight-bearing ability. Made of hardened stainless steel, this implant is fundamentally different from the titanium precursor and allows patients to bear 150–250 lb of weight, a 400% increase in the weight-bearing tolerance of PRECICE^®^ nails. The effect of this stiffer material on bone regenerate formation and consolidation time is unknown.

Aside from weight-bearing potential, differences between these two internal lengthening nails are not well documented. Moreover, reports describing outcomes of femoral lengthening using the STRYDE^®^ MILN are sparse. The aims of this study are to report outcomes of our experience with the STRYDE^®^ nail and to compare these outcomes with PRECICE^®^ MILN for femoral lengthening. Our null hypothesis was that there would be no difference in the 6-months post-operative Limb Deformity-Scoliosis Research Society (LD-SRS) Score [18] when using STRYDE^®^ MILN vs RECICE^®^ MILN for femoral lengthening.

## Methods

### Study design

This is an Institutional Review Board (IRB) approved, single-center, level 3 retrospective cohort study. The study reflects a change in practice with earlier patients receiving the PRECICE^®^ MILN and later patients the STRYDE^®^ MILN as it became available for use.

### Setting

Academic Level-1 referral center, medical records from January 2017 to March 2020 were reviewed to collect data of femoral lengthening procedure using either STRYDE^®^ or PRECICE^®^ MILN.

### Participants

Patients who underwent femoral lengthening using a STRYDE^®^ MILN with a lengthening goal of 8 cm, no associated deformity and no medical co-morbidities were included. PRECICE^®^ MILN cases were matched for age (± 2 years), lengthening goal (± 1 cm) and etiology to maximize homogeneity between groups.

### Intervention and surgical technique

Aside from implant used, both cohorts underwent identical surgical procedures (all surgeries were performed by the 2 senior authors SRR and ATF) including pre-operative templating, vent hole creation at the osteotomy site, flexible reaming 2 mm greater than the nail diameter, percutaneous corticotomy with an osteotome, and iliotibial band release at the distal locking screw insertion site. Post-operatively distraction was started on post-op day 7 at 0.8 mm/day (divided over 4 increments), patients were followed up every two weeks during the distraction phase, where the rate of distraction could be changed based on the radiographic quality of the regenerate bone, then every month during the consolidation phase. Measurement of distraction gap was done on femur radiographs and using the nail as the calibration mark, while measurements of lateral distal femoral angle (LDFA) were done on long leg films. All radiographic measurements were done by the 2 senior authors (SRR and ATF). In the STRYDE^®^ group if the patient’s weight was below the implant weight limit (150–250 lb), they were allowed to be weight bearing as tolerated, while in the PRECICE^®^ group post-operative weight bearing was limited to the manufacturer-recommended implant weight limit (50–75 lb) until regenerate if fully consolidated.

### Outcomes and sample size

The 6-months post-operative LD-SRS Score was selected as the primary outcome measure, it was also collected pre-operatively.

Secondary outcome measures were lengthening achieved, nail size, distraction rate, bone healing index (BHI), adjacent joints loss of range of motion (measured using a goniometer and performed by the treating surgeon, namely SRR or ATF), lateral distal femoral angle (LDFA) and complications. Date of bone union was recorded when 3 of 4 cortices were bridging at the lengthening site using radiographs and as reviewed by the 2 senior authors SSR and ATF who were the treating surgeons.

The LD-SRS questionnaire (supplement 1) is composed of 30 questions that covers 5 domains (supplement 2) namely; pain, function, self-image, mental health and satisfaction with treatment. Each question’s answer gets a score from 1(worst) to 5 (best), the sum score of all answers is then divided by the number of answered questions to yield a final score from 1(worst) to 5 (best).

We chose the improvement limit Δ (minimal clinically important difference, MCID) as 10% (0.5 point), as the possible finding of a smaller detectable difference would not be of clinical significance. In our pretest sample-size analysis, we used a standard deviation (SD) of LDR-SRS score of 0.6 points based on previous report in the literature (18). With a significance level of 5% (two-sided confidence interval of 95%), and a power of 90%, the sample size was calculated to include at least 30 in each group.

### Statistical methods

The statistical analysis was performed using the statistical package SAS statistical software (SAS Institute Inc, Cary, NC, USA).

Mean and range were used to express continuous data while frequency (count) and relative frequency (percentage) was used to express categorical data.

Continuous variables were age, nail size, lengthening achieved, distraction rate, bone healing index (BHI), range of motion, LDFA and LD-SRS score. Categorical variables were gender, laterality, and complication.

The statistical difference was calculated using two-sided, independent sample Student’s *t* test for continuous variables which followed a normal distribution and Mann–Whitney *U* test for those not following a normal distribution. For comparing categorical data, the Chi-squared (*χ*^2^) test was performed for frequencies > 5, while Fisher exact test was performed for frequencies ≤ 5.

Statistical significance was set at alpha (*p *value) ≤ 0.05.

### Ethics, funding, and potential conflicts of interest

This study was approved by the institutional review board (IRB). Authors state that there is no conflict of interest. No benefits in any form have been received or will be received from a commercial party related directly or indirectly to the subject of this article. No funds were received in support of this study.

## Results

**Fig. 1:**
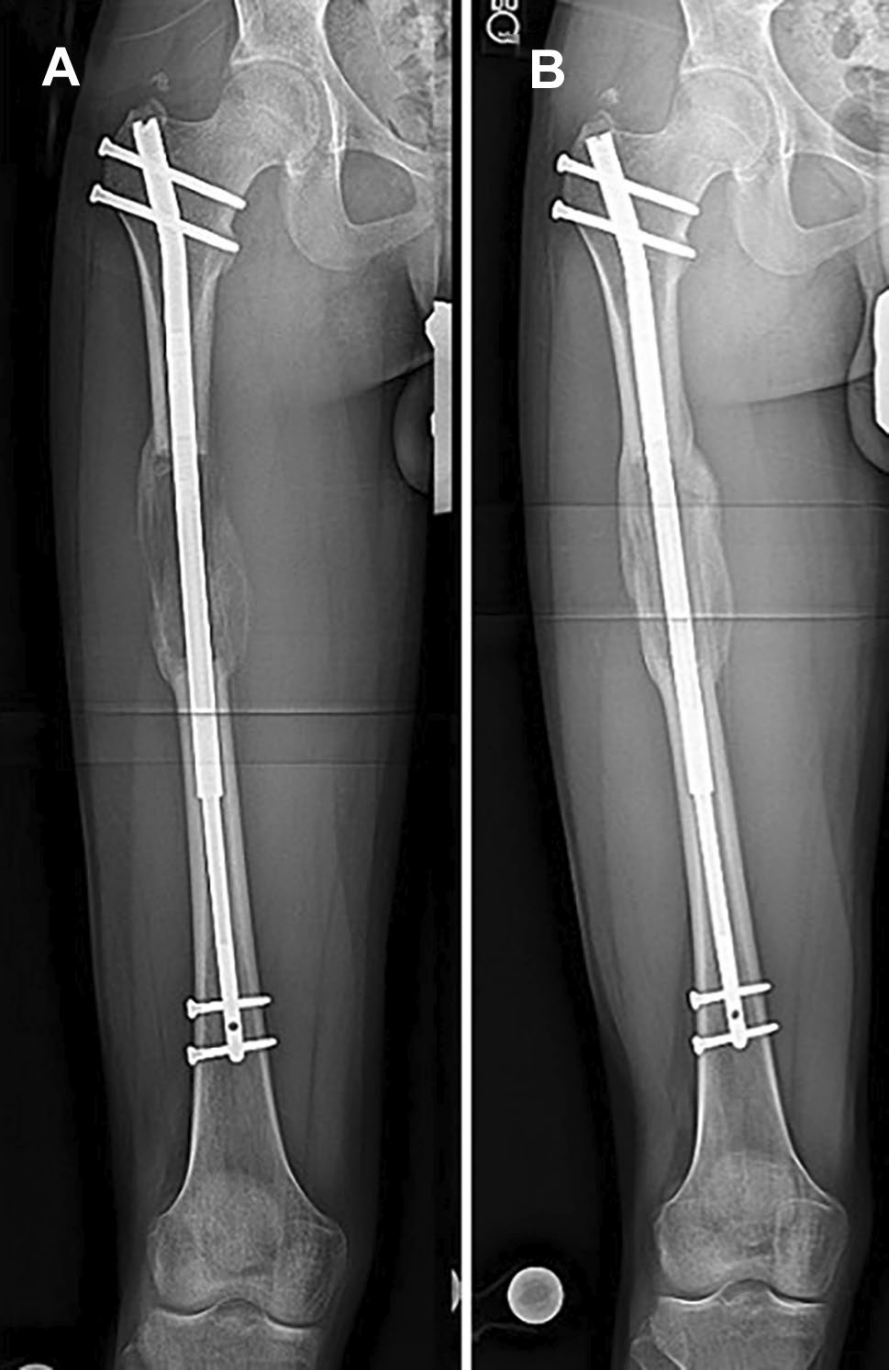
Case example for STRYDE^®^ femoral lengthening, **a** antero-posterior (AP) radiographs at end of distraction, **b** AP radiographs at full consolidation

This was a homogenous group of patients in terms of age, gender, lengthening achieved, and nail size (Table 1). Data review yielded 30 femoral STRYDE^®^ procedures in 16 patients (14 patients with short stature who underwent bilateral femoral lengthening and 2 patients with post-traumatic physeal arrest who underwent unilateral femoral lengthening) who all met the inclusion criteria and were included (Fig. 1).The review also yielded 30 patients who underwent PRECICE^®^ procedures after matching for the STRYDE^®^ group, 18 patients were included (all underwent bilateral femoral lengthening, 17 patients had short stature and 1 patient had Prader–Willi syndrome). Follow up was 14 (12–30) months, no patients were lost to follow-up.

**Table 1:** Patients’ Demographics

	STRYDE	PRECICE	*p* value
Age (years)	31 (17–65)	33 (19–54)	0.46
Gender (F/M)	2 F, 14 M	3 F, 15 M	0.99
Lengthening done (cm)	7.1 (4.1–8)	6.8 (4–8)	0.51
Nail size (mm)	11.5 (10–13)	10.7 (8.5–12.5)	0.14

Patients’ age was 31 (17–54) years and 33 (19–54) years for the STRYDE^®^ and PRECICE^®^ groups, respectively (*p* value = 0.5). Both groups had majority male patients, (87.5% males and 12.5% females in the STRYDE^®^ group compared to 83.3% males and 16.7% females in the PRECICE^®^ group) (*p* value = 0.1). Average lengthening achieved was 7.1 (4.1–8) cm and 6.8 (4–8) cm for the STRYDE^®^ and PRECICE^®^ groups, respectively (*p* value = 0.5). The most frequently nail size used was the 11.5 (10–13) mm and 10.7 (8.5–12.5) mm for the STRYDE^®^ and PRECICE^®^ groups, respectively (*p* value = 0.1).

Distraction rate was 0.6 (0.4–0.9) mm/day and 0.9 (0.6–1) mm/day for the STRYDE^®^ and PRECICE^®^ groups, respectively, the difference was found to be statistically significant (*p* value = 0.003). Bone healing index (BHI) was 0.84 (0.53–1.5) month/cm and 0.67 (0.44–1.2) month/cm for the STRYDE^®^ and PRECICE^®^ groups, respectively, the difference was found to be statistically significant (*p* value = 0.04).

The pre-operative LD-SRS score was 3.9 (3.2–4.8) and 4 (2.9–4.6) in the STRYDE^®^ and PRECICE^®^ groups, respectively (*p* value = 0.09). The 6-months post-operative LD-SRS score was 4.1 (3.5–4.9) and 4.4 (3.8–4.8) for the STRYDE^®^ and PRECICE^®^ groups, respectively (*p* value = 0.07). Change in LD-SRS score (comparing the 6-months score to the pre-op score to assess improvement) was 0.2 (range: 1 improvement–1 worsening) in the STRYDE^®^ group compared to 0.4 (range 0.78 improvement—0.47 worsening) in the PRECICE^®^ group (*p* value = 0.5).

With regard to loss of ROM for adjacent joints, there were no significant differences between groups. Loss of hip ROM was reported following 2 STRYDE^®^ lengthening procedures (15º and 30º), while there was no loss of hip ROM following any of the PRECICE^®^ lengthening procedures. Loss of knee ROM was reported following 2 STRYDE^®^ lengthening procedures (5º and 10º) and following 2 PRECICE^®^ lengthening procedures (5º and 10º).

With regards to the LDFA, the pre-operative LDFA was 87.4° (85–93) and 87.4° (84–93) for the STRYDE^®^ and PRECICE^®^ groups, respectively (*p* value = 0.33). The post-operative LDFA was 88.8° (84–94) and 87.8° (84–92) for the STRYDE^®^ and PRECICE^®^ groups, respectively (*p* value = 0.09). the change in LDFA was 1.6° varus (3 valgus–5 varus) and 0.25° varus (5 valgus–4 varus) for the STRYDE^®^ and PRECICE^®^ groups, respectively (*p* value = 0.08).

Table 2 summarizes patients’ results.

**Table 2:** Results

	STRYDE	PRECICE	*p* value
Distraction rate (mm/day)	0.6 (0.4–0.9)	0.9 (0.6–1)	0.003*
Bone healing index (month/cm)	0.84 (0.53–1.5)	0.67 (0.44–1.2)	0.04*
Pre-op LD-SRS score	3.9 (3.2–4.8)	4 (2.9–4.6)	0.09
Post-op LD-SRS score	4.1 (3.5–4.9)	4.4 (3.8–4.8)	0.07
Change in LD-SRS score	0.2 (1 improvement—1 worsening)	0.4 (0.8 improvement—0.5 worsening)	0.50
Pre-op LDFA (degrees)	87.4 (85–93)	87.4 (84–93)	0.33
Post-op LDFA (degrees)	88.8 (84–94)	87.8 (84–92)	0.09
Change in LDFA (degrees)	1.6 varus (3 valgus—5 varus)	0.25 varus (5 valgus—4 varus)	0.08

### Complications

We used the Black et al. [19] classification of complications, type I complications (minimal intervention required; treatment goal still achieved e.g. mild joint contractures); type II complications (substantial change in treatment plan but treatment goal still achieved e.g. return to OR for BMAC, exchange nailing); type IIIA complications (failure to achieve treatment goal e.g. aborted lengthening); type IIIB complications (new pathology e.g. joint subluxation, loss of ROM or deep infection).

**Fig. 2:**
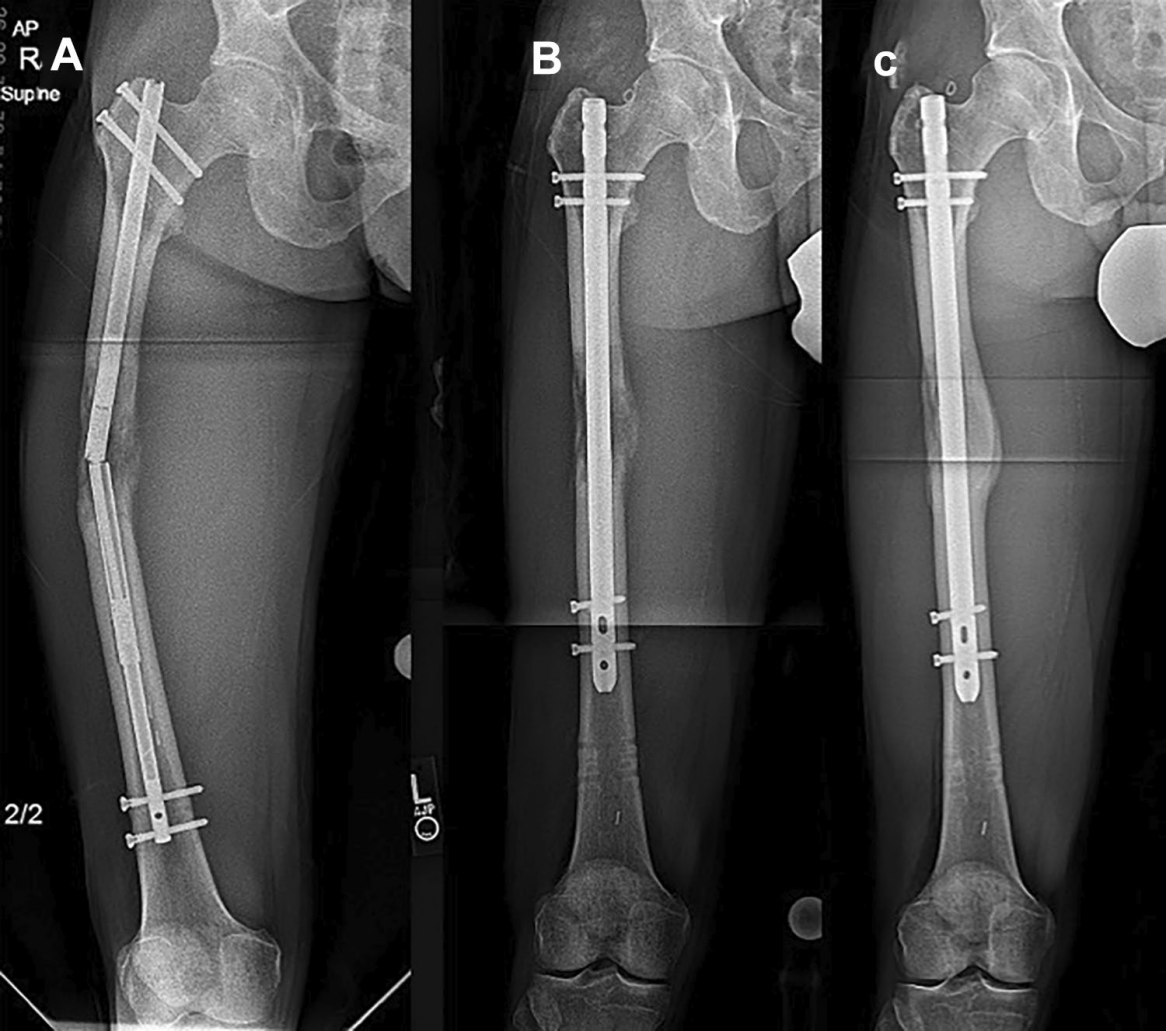
Case example for PRECICE^®^ femoral lengthening with implant failure requiring return to the operating room, **a** antero-posterior (AP) radiographs showing catastrophic nail failure, **b** AP radiographs after exchange nailing, **c** AP radiographs at full consolidation

In the PRECICE^®^ group, 4 nails had type II complication: 3 femora had mechanical nail failure during the consolidation phase and required exchange nailing (Fig. 2) and 1 femur showed delayed consolidation that necessitated Bone Marrow Aspirate Concentrate (BMAC) injection. 3 other femora had type IIIA complications in the form of “crown breakage” when they have achieved more than 7 cm of length but less than the lengthening goal of 8 cm.

**Fig. 3:**
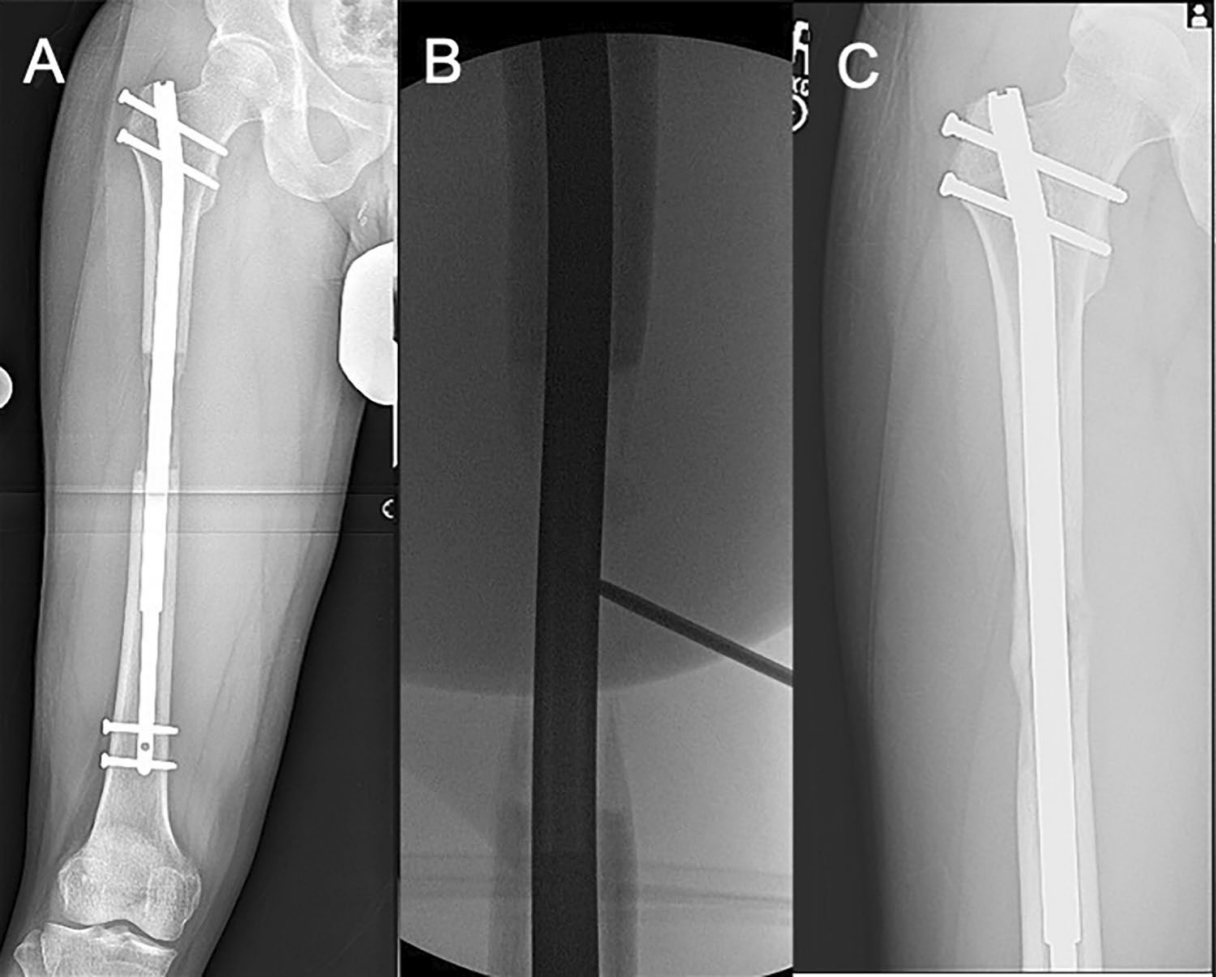
Case example for STRYDE^®^ femoral lengthening necessitating BMAC injection, **a** antero-posterior (AP) radiographs at showing inadequate regenerate quality, **b** intra-op fluoroscopy image performing BMAC injection, **c** AP radiographs at full consolidation

In the STRYDE^®^ group (Fig. 3) 4 femora had type II complication in the form of delayed consolidation that necessitated BMAC injection. No patients in the STRYDE^®^ group had mechanical nail complications.

No patients suffered from nonunion, VTE, or infection.

## Discussion

The Ilizarov apparatus is widely accepted as the gold standard for limb lengthening surgery. External fixators are associated with a high risk of pin tract infections, risk of fractures during and after lengthening, and soft tissue tethering [5]. They present the patient with physical and psychosocial obstacles, as there are low levels of tolerance with maneuvering the bulky device. The objective of distraction osteogenesis can be achieved in a more manageable fashion with greater ease and less complications when using intramedullary lengthening. As completely internal devices, they avoid many of the complications associated with external fixators. MILNs provide more comfort and have overall better patient satisfaction. However, limited data exists detailing outcomes of modern MILNs.

When the PRECICE^®^ MILN [11–22] was first introduced in 2011, it took internal lengthening nailing to new heights as a telescoping rod that used an actuator drive mechanism to distract the titanium nail. It allowed surgeons to control distraction using an external remote with two revolving magnets that communicated with the magnet in the nail. The explicit control over lengthening that the PRECICE^®^ nail provides, made early internal bone lengthening devices with mechanically activated nails such as the Albizzia^®^ and ISKD^®^ obsolete. Due to their design, these nails lacked control of the rate and rhythm of distraction, which resulted in complications such as premature consolidation, neurovascular injury, and nonunion [7–9].

The introduction of the STRYDE^®^ MILN [23] in 2018 further improved MILN technology by building upon the reliable performance of its PRECICE^®^ predecessor. It is made of steel and permits larger weight-bearing loads. The PRECICE^®^ nail only allows for 50–75 lb (22–34 kg) of weight-bearing [24], which prevents patients from returning to activities of daily living in a timely manner. The STRYDE^®^ nail allows for 150–250 lb (68–113 kg) of weight, which maximizes healing potential with the addition of axial compression.

Based on the findings from this study, there was no significant functional difference based on the patient-reported clinical scores. However, distraction rates were slower for STRYDE^®^ patients, and the BHI was higher. A higher BHI indicates this procedure is associated with slower healing rates, as BHI is defined as time from osteotomy and surgical implantation of the nail to time of bone union. This confirms surgeon perceptions that the bone heals slower with the STRYDE^®^ implant. Surgeons elected a slower lengthening speed based on radiographic feedback at patient visits which led to a prolonged distraction period and no change in the consolidation period effecting a slower BHI. In addition, a handful of femora in the sample required BMAC which is a biological stimulant rich in mononuclear cells that promotes osteogenesis [25]. There were more patients requiring BMAC to enhance bone regeneration and healing in the STRYDE^®^ group which reflected that despite using a slower rate of lengthening, there was still an increase in prolonged consolidation requiring intervention.

The STRYDE^®^ MILN has the advantage of greater weight-bearing potential for patients. This comes at the cost of time necessary for strong bone consolidation given the higher BHI and higher incidence of cases requiring BMAC in the STRYDE^®^ group. Nonetheless, the weight-bearing potential of this nail has the theoretical benefit of axial compression, which may promote bone healing, although this study did not find any evidence of this advantage. Therefore, treatment plans should be tailored to the specific needs of each patient, as there is a sliding scale of risk and benefit. The risk of slower healing may be outweighed by the benefit of early full weight bearing and a rapid return to work, for example. The STRYDE^®^ has a great benefit of mechanical superiority. PRECICE^®^ had 6 mechanical complications in this series compared with none in the STRYDE^®^ group. This reflects the inability of patients undergoing bilateral femur surgery to adhere to the strict weight-bearing precautions required by PRECICE^®^.

This retrospective study has several limitations. The sample size was not big enough to allow for statistical comparison of the complications that are infrequent, the comparison was not contemporaneous nor was it randomized, and the outcome measures have subjective elements. Since not all PRECICE^®^ MILN were included, there may be a selection bias. There was a lack of long-term follow-up. These demerits could be acceptable considering the fact that the surgery is not very common, and the implants have been in the market only for the last few years.

This data should not be applied to tibial STRYDE^®^ as this bone was not studied.

## Conclusion

The STRYDE^®^ MILN yields comparable clinical scores and functional results to those of PRECICE^®^ MILN. We found two main advantages to using the STRYDE^®^ MILN; namely, fewer mechanical nail complications and the fact that patients with STRYDE^®^ IMLNs were permitted to be fully weight bearing allowed them to resume activities of daily living more quickly compared to patients with PRECICE^®^ MILNs. However, STRYDE MILNs are associated with slower healing rate (larger BHI), which explains surgeons’ tendency to distract at a slower rate when using this device.

## Supplementary Information

Below is the link to the electronic supplementary material.

Supplementary file1 (PDF 273 KB)

Supplementary file2 (PDF 106 KB)
