# Supplementary material for: STRYDE versus PRECICE magnetic internal lengthening nail for femur lengthening
Source: Arch Orthop Trauma Surg. 2021 May 13;142(12):3555–61. doi: 10.1007/s00402-021-03943-8 (PMC9596511; doi:10.1007/s00402-021-03943-8)
Supplement: Supplementary file 1 — Supplementary file1 (PDF 273 KB) [file 402_2021_3943_MOESM1_ESM.pdf]

The Limb Deformity Modified SRS (LD-SRS) Score

Examination: ☐Pre-treatment ☐3 mos. ☐6 mos. ☐1 year ☐2 years

In the following questionnaire, the term "limb" refers to the body part currently being treated, including the joint above it and the joint below it. If more than one body part is involved in the current treatment plan, please fill out a separate questionnaire for each one.

Side: ☐Right ☐Left

Body Part: ☐Upper arm ☐Forearm ☐Hand/Wrist
☐Thigh ☐Lower leg ☐Foot/Ankle

Please select the one best answer to each question unless otherwise indicated. If you already have had surgery, please complete sections 1 and 2. Otherwise, just complete section 1.

All results will be kept confidential

Section 1: All patients

1. Which one of the following best describes the amount of limb pain you have experienced during the past 6 months?

- ☐ None
- ☐ Mild
- ☐ Moderate
- ☐ Moderate to severe
- ☐ Severe

2. Which one of the following best describes the amount of limb pain you have experienced over the last month?

- ☐ None
- ☐ Mild
- ☐ Moderate
- ☐ Moderate to severe
- ☐ Severe

3. During the past 6 months have you been a very nervous person?

- ☐ None of the time
- ☐ A little of the time
- ☐ Some of the time
- ☐ Most of the time
- ☐ All of the time

4. If you had to spend the rest of your life with your limb shaped as it is right now, how would you feel about it?

- ☐ Very happy
- ☐ Somewhat happy
- ☐ Neither happy nor unhappy
- ☐ Somewhat unhappy
- ☐ Very unhappy

5. What is your current level of activity?

- ☐ Full activities without restriction
- ☐ Moderate manual labor and moderate sports, such as walking and biking
- ☐ Primarily no activity
- ☐ Light labor, such as household chores
- ☐ Bedridden/wheelchair

6. How do you look in clothes?

- ☐ Very good
- ☐ Good
- ☐ Fair
- ☐ Bad
- ☐ Very bad

7. In the past 6 months have you felt so down in the dumps that nothing could cheer you up?

- ☐ Never
- ☐ Rarely
- ☐ Sometimes
- ☐ Often
- ☐ Very often

8. Do you experience limb pain when at rest?

- ☐ Never
- ☐ Rarely
- ☐ Sometimes
- ☐ Often
- ☐ Very often

9. What is your current level of work/school activity?

- ☐ 100% normal
- ☐ 75% normal
- ☐ 50% normal
- ☐ 25% normal
- ☐ 0% normal

10. Which of the following best describes the appearance of your limb:

- ☐ Very good
- ☐ Good
- ☐ Fair
- ☐ Bad
- ☐ Very bad

11. Which one of the following best describes your medication usage for your limb?

- ☐ None
- ☐ Non-narcotics weekly or less (e.g., Tylenol, Ibuprofen)
- ☐ Non-narcotics daily
- ☐ Narcotics weekly or less (e.g., Percocet, Lorcet, Codeine, Darvocet)
- ☐ Narcotics daily
- ☐ Other (please specify below)

  Medication:

  Usage (weekly or less or daily)

12. Does your limb limit your ability to do things around the house?

- ☐ Never
- ☐ Rarely
- ☐ Sometimes
- ☐ Often
- ☐ Very often

13. Have you felt calm and peaceful during the past 6 months?

- ☐ All of the time
- ☐ Most of the time
- ☐ Some of the time
- ☐ A little of the time
- ☐ None of the time

14. Do you feel that your limb condition affects your personal relationships?

- ☐ None
- ☐ Slightly
- ☐ Mildly
- ☐ Moderately
- ☐ Severely

15. Are you and/or your family experiencing financial difficulties because of your limb?

- ☐ None
- ☐ Slightly
- ☐ Mildly
- ☐ Moderately
- ☐ Severely

16. In the past 6 months have you felt downhearted and blue?

- ☐ Never
- ☐ Rarely
- ☐ Sometimes
- ☐ Often
- ☐ Very often

17. In the last 3 months have you taken any sick days from work/school due to limb pain and, if so, how many?

- ☐ 0
- ☐ 1
- ☐ 2
- ☐ 3
- ☐ 4 or more

18. Do you go out more or less than your friends?

- ☐ Much more
- ☐ More
- ☐ Same
- ☐ Less
- ☐ Much less

19. Do you feel attractive with your current limb condition?

- ☐ Yes, very
- ☐ Yes, somewhat
- ☐ Neither attractive nor unattractive
- ☐ No, not very much
- ☐ No, not at all

20. Have you been a happy person during the past 6 months?

- ☐ All of the time
- ☐ Most of the time
- ☐ Some of the time
- ☐ A little of the time
- ☐ None of the time

Section 2: After Completion of Treatment

21. Are you satisfied with the results of your limb management?
- ☐ Very satisfied
- ☐ Satisfied
- ☐ Neither satisfied nor unsatisfied
- ☐ Unsatisfied
- ☐ Very unsatisfied

22. Would you have the same management again if you had the same condition?
- ☐ Definitely yes
- ☐ Probably yes
- ☐ Not sure
- ☐ Probably not
- ☐ Definitely not

23. On a scale of 1 to 9, with 1 being very low and 9 being extremely high, how would you rate your self-image?

☐1 ☐2 ☐3 ☐4 ☐5 ☐6 ☐7 ☐8 ☐9

24. Compared with before treatment, how do you feel you now look?
- ☐ Much better
- ☐ Better
- ☐ Same
- ☐ Worse
- ☐ Much worse

25. Has your limb treatment changed your function and daily activity?
- ☐ Much better
- ☐ Better
- ☐ Same
- ☐ Worse
- ☐ Much worse

26. Has your limb treatment changed your ability to enjoy sports/hobbies?

- ☐ Much better
- ☐ Better
- ☐ Same
- ☐ Worse
- ☐ Much worse

27. How has your limb treatment changed your limb pain?

- ☐ Much better
- ☐ Better
- ☐ Same
- ☐ Worse
- ☐ Much worse

28. Has your treatment changed your confidence in personal relationships with others?

- ☐ Much better
- ☐ Better
- ☐ Same
- ☐ Worse
- ☐ Much worse

29. Has your treatment changed the way others view you?

- ☐ Much better
- ☐ Better
- ☐ Same
- ☐ Worse
- ☐ Much worse

30. Has your treatment changed your self-image?

- ☐ Much better
- ☐ Better
- ☐ Same
- ☐ Worse
- ☐ Much worse

Please mark on the drawings any areas where you feel pain. If you are not having any pain, leave blank and initial.

Use the following key to show particular types of pain

KEY:

Pins & needles = 000000

Burning = XXXXXX

Stabbing = I//I/I

Deep ache = ZZZZZZ

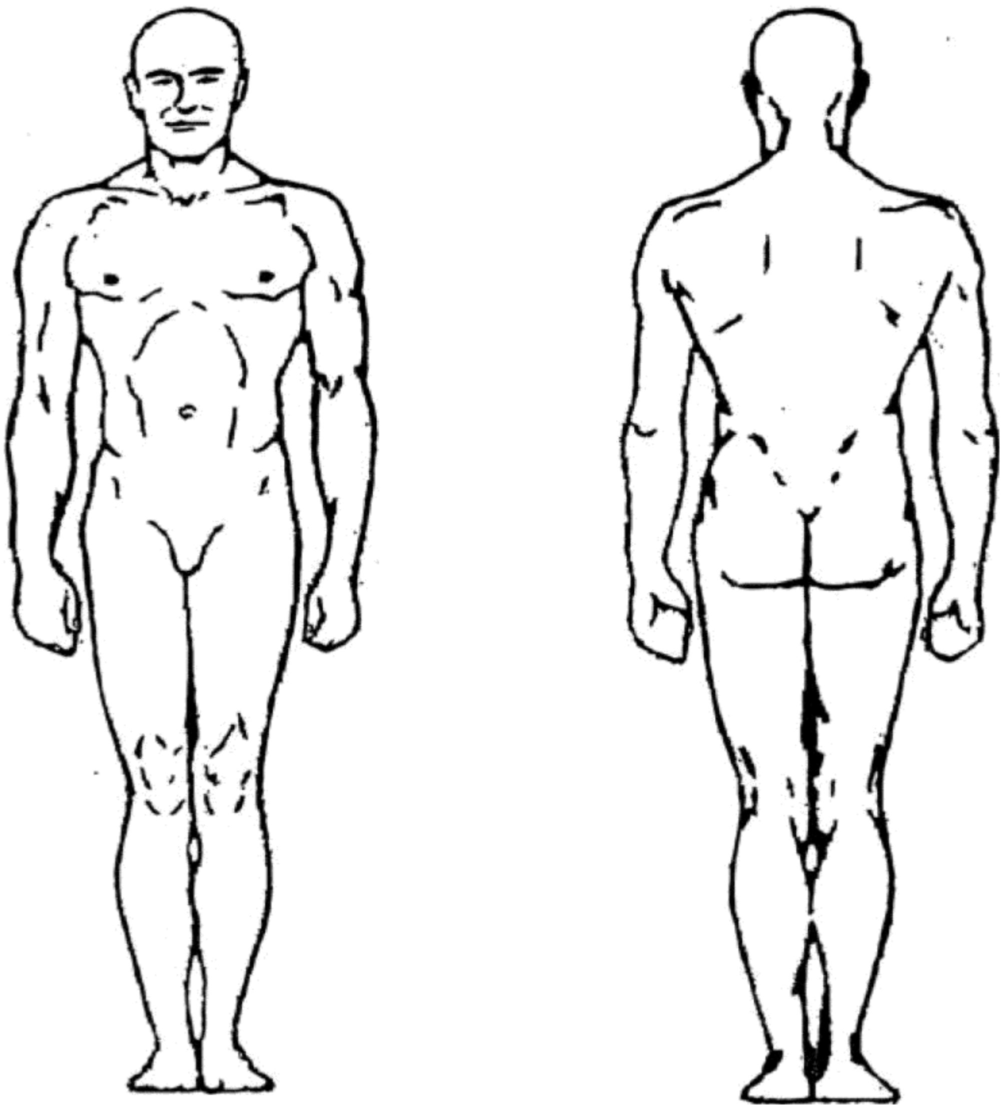

LD-SRS Patient Questionnaire:

Patient Name: ______________________________ **Age:** ___________ **Date:** ______________

Medical Record #: ______________________ **SS#:** _______________________ **Sex:** _____

Exam: Pre-treatment 3 mos. 6 mos. 1 year ___ years

Your doctors are carefully evaluating the condition of your limb before and after your treatment. Please circle the one best answer to each question unless otherwise indicated. If you already have had surgery, please complete **sections 1** and **2**; otherwise, just complete section **1**.

All results will be kept confidential.

Section 1: All Patients

1. Which one of the following best describes the amount of limb pain you have experienced during the past 6 months?

- ☐ None 5
- ☐ Mild 4
- ☐ Moderate 3
- ☐ Moderate to severe 2
- ☐ Severe 1

2. Which one of the following best describes the amount of limb pain you have experienced over the last month?

- ☐ None 5
- ☐ Mild 4
- ☐ Moderate 3
- ☐ Moderate to severe 2
- ☐ Severe 1

3. During the past 6 months have you been a very nervous person?

- ☐ None of the time 5
- ☐ A little of the time 4
- ☐ Some of the time 3
- ☐ Most of the time 2
- ☐ All of the time 1

4. If you had to spend the rest of your life with your limb shape as it is right now, how would you feel about it?

- ☐ Very happy 5
- ☐ Somewhat happy 4
- ☐ Neither happy nor unhappy 3
- ☐ Somewhat unhappy 2
- ☐ Very unhappy 1

5. What is your current level of activity?

- ☐ Bedridden/Wheelchair 1
- ☐ Primarily no activity 2
- ☐ Light labor, such as household chores 3
- ☐ Moderate manual labor and moderate 4 sports, such as walking and biking
- ☐ Full activities without restriction 5

6. How do you look in clothes?

- ☐ Very good 5
- ☐ Good 4
- ☐ Fair 3
- ☐ Bad 2
- ☐ Very bad 1

7. In the past 6 months have you felt so down in the dumps that nothing could cheer you up?

- ☐ Very often 1
- ☐ Often 2
- ☐ Sometimes 3
- ☐ Rarely 4
- ☐ Never 5

8. Do you experience limb pain when at rest?

- ☐ Very often 1
- ☐ Often 2
- ☐ Sometimes 3
- ☐ Rarely 4
- ☐ Never 5

9. What is your current level of work/ school activity?

- ☐ 100% normal 5
- ☐ 75% normal 4
- ☐ 50% normal 3
- ☐ 25% normal 2
- ☐ 0% normal 1

10. Which of the following best describes the appearance of your limb

- ☐ Very Good 5
- ☐ Good 4
- ☐ Fair 3
- ☐ Poor 2
- ☐ Very Poor 1

11. Which one of the following best describes your medication usage for your limb?

- ☐ None 5
- ☐ Non-narcotics weekly or less (e.g., Tylenol, Ibuprofen) 4
- ☐ Non-narcotics daily 3
- ☐ Narcotics weekly or less (e.g., Tylenol #3, Lorocet, Percocet, Darvocet) 2
- ☐ Narcotics daily 1
- ☐ Other (*please specify below*)

Medication:

____

Usage (weekly or less or daily):

12. **Does your limb limit your ability to do things around the house?**

☐ Never 5 ☐ Often 2
☐ Rarely 4 ☐ Very Often 1
☐ Sometimes 3

13. **Have you felt calm and peaceful during the past 6 months?**

☐ All of the time 5 ☐ A little of the time 2
☐ Most of the time 4 ☐ None of the time 1
☐ Some of the time 3

14. **Do you feel that your limb condition affects your personal relationships?**

☐ None 5 ☐ Moderately 2
☐ Slightly 4 ☐ Severely 1
☐ Mildly 3

15. **Are you and or your family experiencing financial difficulties because of your limb?**

☐ Severely 1 ☐ Slightly 4
☐ Moderately 2 ☐ None 5
☐ Mildly 3

16. **In the past 6 months have you felt down hearted and blue?**

☐ Never 5 ☐ Often 2
☐ Rarely 4 ☐ Very Often 1
☐ Sometimes 3

17. **In the last 3 months have you taken any sick days from work/school due to limb pain, and if so, how many?**

☐ 0 5 ☐ 1 4 ☐ 2 3 ☐ 3 2 ☐ 4 or more 1

18. **Do you go out more or less than your friends?**

☐ Much More 5 ☐ Less 2
☐ More 4 ☐ Much less 1
☐ Same 3

19. **Do you feel attractive with your current back condition?**

☐ Yes, very 5 ☐ No, not very much 2
☐ Yes, somewhat 4 ☐ No, not at all 1
☐ Neither attractive nor unattractive 3

20. **Have you been a happy person during the past 6 months?**

☐ None of the time 1 ☐ Most of the time 4
☐ A little of the time 2 ☐ All of the time 5
☐ Some of the time 3

21. **Are you satisfied with the results of your limb management?**

☐ Very satisfied 5 ☐ Unsatisfied 2
☐ Satisfied 4 ☐ Very unsatisfied 1
☐ Neither satisfied nor unsatisfied 3

22. **Would you have the same management again if you had the same condition?**

☐ Definitely yes 5 ☐ Probably not 2
☐ Probably yes 4 ☐ Definitely not 1
☐ Not sure 3

23. **On a scale of 1 to 9, with 1 being very low and 9 being extremely high, how would you rate your self-image?**

☐1 ☐2	☐3 ☐4	☐5	☐6 ☐7	☐8 ☐9
1	2	3	4	5

Section 2: Post-surgery patients only

24. **Compared with before treatment, how do you feel you now look?**

☐ Much Better 5 ☐ Worse 2
☐ Better 4 ☐ Much worse 1
☐ Same 3

25. **Has your limb treatment changed your function and daily activity?**

☐ Much Better 5 ☐ Worse 2
☐ Better 4 ☐ Much worse 1
☐ Same 3

26. **Has your limb treatment changed your ability to enjoy sports/hobbies?**

☐ Much Better 5 ☐ Worse 2
☐ Better 4 ☐ Much worse 1
☐ Same 3

27. **Has your limb treatment changed your limb pain?**

☐ Much Better 5 ☐ Worse 2
☐ Better 4 ☐ Much worse 1
☐ Same 3

28. **Has your treatment changed your confidence in personal relationships with others?**

☐ Much Better 5 ☐ Worse 2
☐ Better 4 ☐ Much worse 1
☐ Same 3

29. **Has your treatment changed the way others view you?**

☐ Much Better 5 ☐ Worse 2
☐ Better 4 ☐ Much worse 1
☐ Same 3

30. **Has your treatment changed your self-image**

☐ Much Better 5 ☐ Worse 2
☐ Better 4 ☐ Much worse 1
☐ Same 3
