# Supplementary material for: STRYDE versus PRECICE magnetic internal lengthening nail for femur lengthening
Source: Arch Orthop Trauma Surg. 2021 May 13;142(12):3555–61. doi: 10.1007/s00402-021-03943-8 (PMC9596511; doi:10.1007/s00402-021-03943-8)
Supplement: Supplementary file 2 — Supplementary file2 (PDF 106 KB) [file 402_2021_3943_MOESM2_ESM.pdf]

LD-SRS Patient Questionnaire/Score Sheet

Name: ____________________________ Today's Date: ______________
Mo Day Year

Age: ______ __________ Sex: M F
Yr Mo

Diagnoses: ______________________ Deformity/Side______________ ______________
____
____

Date Initiated: ____ ____ ____ Follow-up _____ _____
____ ____ ____ _____ _____
____ ____ ____ _____ _____
Mo Day Yr Yrs Mo

**DOMAIN**	(Score: 5 Best – 1 Worst)	Post-Surgery Questions	Score Pt/Possible (Max) **A**	#Questions Answered(Possible) **B**	Mean Score *** **A÷B**
Function/ Activity	____ ____ ____ ____ ____ 5* 9 12 15 18	____ ____ 25 26	____(___)(25) (35)+	____(5) (7)+	____
Pain	____ ____ ____ ____ ____ 1 2 8 11 17	____ 27	____(___)(25) (30)	____(5) (6)	____
Self-Image/ appearance	____ ____ ____ ____ ____ ____ 4 6 10 14 19 23	____ ____ ____ 28 29 30	____(___)(30) (45)	____(6) (9)	____
Mental health**	____ ____ ____ ____ ____ 3 7 13 16 20		____(___)(25)	____(5)	____
	**SUB TOTAL**		____(___)(105) (135)	____(21) (27)	____
Satisfaction with management	____ ____ 21 22	____ 24	____(___)(10) (15)	____(2) (3)	____
	**TOTAL**		____(___)(115) (150)	____(23) (30)	____

+max/possible with post-surgery questions

***Mean Score
5 Best
1 Worst

*Question Number

**Questions adopted with permission from SF-36

SCORING INSTRUCTIONS

Unanswered questions – reduce questions answered denominator by appropriate number
Delete questions with more than one response
Domain can't be scored if fewer than 3 questions answered
